# Extended-Interval Dosing of Rezafungin against Azole-Resistant Aspergillus fumigatus

**DOI:** 10.1128/AAC.01165-19

**Published:** 2019-09-23

**Authors:** Nathan P. Wiederhold, Laura K. Najvar, Rosie Jaramillo, Marcos Olivo, Brian L. Wickes, Gabriel Catano, Thomas F. Patterson

**Affiliations:** a Department of Pathology and Laboratory Medicine, University of Texas Health Science Center at San Antonio, San Antonio, Texas, USA; b Department of Medicine, Division of Infectious Diseases, University of Texas Health Science Center at San Antonio, San Antonio, Texas, USA; c Department of Microbiology, Immunology, and Molecular Genetics, University of Texas Health Science Center at San Antonio, San Antonio, Texas, USA; d South Texas Veterans Health Care Center, San Antonio, Texas, USA

**Keywords:** *Aspergillus fumigatus*, TR34/L98H, azole resistance, invasive aspergillosis, murine model, rezafungin

## Abstract

We evaluated extended-interval dosing of the investigational echinocandin rezafungin (1, 4, and 16 mg/kg on days 1, 4, and 7 postinoculation) for the treatment of disseminated invasive aspergillosis caused by azole-resistant Aspergillus fumigatus. Survival was significantly improved in mice treated with each dose of rezafungin and supratherapeutic posaconazole (20 mg/kg twice daily). Kidney fungal burden, as measured by quantitative real-time PCR, was also significantly reduced in mice treated with rezafungin although variability was observed.

## Abstract (precis)

We evaluated extended-interval dosing of the investigational echinocandin rezafungin (1, 4, and 16 mg/kg on days 1, 4, and 7 postinoculation) for the treatment of disseminated invasive aspergillosis caused by azole-resistant Aspergillus fumigatus. Survival was significantly improved in mice treated with each dose of rezafungin and supratherapeutic posaconazole (20 mg/kg twice daily).

## TEXT

Growing concern exists for azole-resistant Aspergillus fumigatus due to prolonged exposure to members of this antifungal class in patients with acute invasive or chronic pulmonary aspergillosis, or to environmental exposure of isolates to azoles used in agriculture and various materials to prevent rotting and the growth of molds (1). Azole resistance due to environmental exposure has been associated with mutations within the *CYP51A* gene along with tandem base pair repeats in the promoter region of this gene (i.e., TR_34_/L98H and TR_46_/Y121F/T289A) (2–4). Azole-resistant A. fumigatus isolates with such resistance mechanisms have now been found in numerous countries worldwide (5–7). The rise of azole resistance in A. fumigatus is particularly concerning, since the azoles are the only orally available agents for the treatment of invasive aspergillosis and the duration of therapy is often prolonged. Rezafungin is an investigational echinocandin that demonstrates potent *in vitro* activity against Aspergillus species, including azole-resistant A. fumigatus isolates (8, 9). Structurally, rezafungin is similar to anidulafungin (10), but with the hemiaminal group replaced with a choline aminal ether, resulting in a more stable compound with a prolonged half-life in multiple animal species and humans (half-life of ∼130 h in humans) (10–13). This long half-life and high plasma exposure of rezafungin translate into sustained drug levels and high tissue penetration with less frequent dosing compared with other echinocandins (14). Our objective was to determine if extended-interval dosing of rezafungin would also be effective against disseminated invasive aspergillosis caused by an azole-resistant isolate.

Male ICR mice were rendered neutropenic with intraperitoneal (i.p.) doses of cyclophosphamide and 5-fluorouracil (200 mg/kg and 5 mg/mouse, respectively, the day prior to inoculation). Mice were inoculated intravenously with a clinical A. fumigatus isolate (UTHSCSA DI15-116) harboring a TR_34_/L98H mutation (rezafungin minimum effective concentration [MEC], 0.06 μg/ml; posaconazole MIC, 1 μg/ml) (6). Treatment with vehicle control, rezafungin (1, 4, or 16 mg/kg i.p. on days 1, 4, and 7) or supratherapeutic posaconazole (20 mg/kg orally [p.o.] twice a day [BID]) (15) began 24 h postinoculation and continued through day 7. In the fungal burden arm, mice were humanely euthanized on day 8, and kidneys were collected for fungal burden analysis by CFU enumeration (CFU/g) and quantitative real-time PCR (qPCR) as previously described (16). In the survival arm, mice were followed off therapy until day 12. Fungal burden was also measured as mice became moribund in the survival arm. Survival was assessed by Kaplan-Meier analysis and the log rank test, and fungal burden was assessed by analysis of variance (ANOVA) with Tukey’s posttest for multiple comparisons. The animal protocol was approved by the University of Texas Health Science Center Institutional Animal Care and Use Committee.

**FIG 1:**
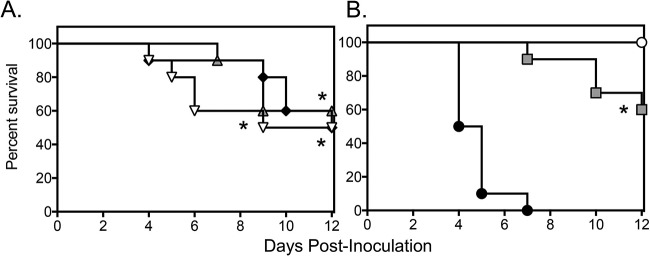
Survival curves in a neutropenic murine model of invasive aspergillosis caused by an azole-resistant A. fumigatus isolate harboring a TR_34_/L98H mutations. One day post intravenous inoculation, mice were treated with extended-interval dosing of rezafungin (1, 4, or 16 mg/kg i.p. on days 1, 4, and 7 postinoculation) (A), vehicle control, or posaconazole (20 mg/kg p.o. BID) (B). Treatment continued through day 7, and mice were followed off therapy until day 12. Black circles, vehicle control; gray squares, posaconazole; white circle, uninfected control (to assess for bacterial superinfections); inverted white triangle, rezafungin 1 mg/kg; black diamond, rezafungin 4 mg/kg; gray triangle, rezafungin 16 mg/kg. *N* = 10 mice per group; lines represent mean values. *, *P* < 0.05 versus vehicle control.

**FIG 2:**
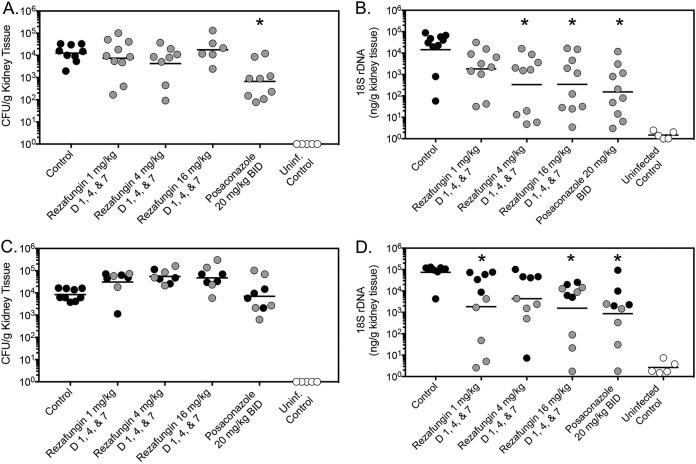
Fungal burden in neutropenic mice with invasive aspergillosis caused by an azole-resistant A. fumigatus isolate harboring a TR_34_/L98H mutations. Fungal burden was measured by CFU enumeration (CFU/g) (A) and quantitative real-time PCR (ng/g DNA) (B), measured on day 8 postinoculation in the fungal burden arm, and on day 12 postinoculation (C) or as the mice became moribund in the survival arm (D). Black circles represent mice that were moribund and humanely euthanized prior to the day 12 time point in the survival arm (C and D), while gray circles represent infected mice that survived to this time point. *, *P* value < 0.05 versus vehicle control. D, day; rDNA, ribosomal DNA.

Extended-interval dosing of rezafungin was associated with a survival advantage in this experimental model of disseminated aspergillosis. Both median survival and percent survival were significantly improved at each dosage level of rezafungin (median survival range, 10.5 to 12 days; percent survival, 50% to 60%) compared to vehicle control (4.5 days and 0%; *P* ≤ 0.0325 for all comparisons) (Fig. 1A). Survival was also improved in mice administered supratherapeutic posaconazole (>12 days and 60%; *P* < 0.0001 versus vehicle) (Fig. 1B). Changes in fungal burden within kidney tissue were also observed with extended-interval dosing of rezafungin and posaconazole. However, these changes were assay dependent. On day 8 postinoculation, 1 day after therapy stopped, only posaconazole was associated with a significant reduction in CFU (mean, 2.84 log_10_ CFU/g) compared to vehicle control (4.10 log_10_ CFU/g; *P* = 0.0021) (Fig. 2A). In contrast, when measured by qPCR, extended-interval doses of rezafungin of 4 mg/kg and 16 mg/kg (2.53 and 2.54 log_10_ ng/g DNA, respectively) and posaconazole (2.19 log_10_ ng/g DNA) significantly lowered fungal burden compared to vehicle control (4.16 log_10_ ng/g DNA; *P* ≤ 0.0131 for all comparisons) (Fig. 2B). In the survival arm, no treatment group was associated with significant reductions in CFU (Fig. 2C). In contrast, when measured by qPCR, rezafungin 1 mg/kg and 16 mg/kg (3.26 and 3.19 log_10_ ng/g DNA, respectively) and posaconazole (2.94 log_10_ ng/g DNA) reduced fungal burden compared to vehicle control (4.87 log_10_ ng/g DNA; *P* ≤ 0.041 for all comparisons) (Fig. 2D). Overall, the fungal burden results observed with rezafungin and posaconazole as measured by qPCR in the survival arm were consistent with the improvements in survival observed in this study, as 94.4% of treated mice whose fungal burden was above the mean value for all treatment groups (3.36 log_10_ ng/g DNA) succumbed to infection. Similar relationships were not observed when measured by CFU. Others have previously reported the utility of qPCR in assessing changes in fungal burden in animal models of invasive aspergillosis with similar variability (16–18). Interestingly, extended-interval dosing of rezafungin at 4 mg/kg was not associated with a significant reduction in fungal burden in the survival arm, despite improvements in overall survival at this dosage level.

The *in vivo* results of this study are in agreement with the *in vitro* activity previously reported for rezafungin against azole-resistant A. fumigatus and cryptic *Aspergillus* species against which the azoles have reduced activity (8, 9). One study also demonstrated good *in vivo* efficacy of rezafungin with extended-interval dosing in a neutropenic murine model of invasive candidiasis (14). In this previous work, which used the same doses and administration frequency, the rezafungin maximum concentration of drug in serum (*C*_max_) fell between 2.60 μg/ml to 26.5 μg/ml, and the area under the concentration-time curve from 0 h to infinity (AUC_0-inf_) was between 93.2 μg · h/ml and 972 μg · h/ml. The extended-interval dosing used in this previous study and in the current work mimicked the pharmacokinetics achieved with once-weekly regimens studied in humans (13, 14, 19). The results of the current study also suggest that extended-interval dosing of rezafungin may be effective in the treatment of invasive aspergillosis, with improved survival and reduction in fungal burden as measured by qPCR, which is also consistent with activity of echinocandins against *Aspergillus* (17). However, it is unknown if outcomes could be further improved with a different dosing strategy of rezafungin, given the differences observed in echinocandin activity between Candida (fungicidal) and *Aspergillus* (fungistatic) species (20). Both the AUC/MIC and *C*_max_/MIC pharmacokinetic/pharmacodynamic parameters have been associated with improved echinocandin treatment outcomes in experimental models of invasive fungal infections, including invasive aspergillosis (18, 21–24). Extended-interval dosing of rezafungin would be advantageous in the treatment of azole-resistant aspergillosis, as daily intravenous administration of the currently approved echinocandins is problematic given the long durations of therapy often needed in patients with these invasive mycoses. However, further studies are warranted to assess the potential benefits of extended-interval dosing of rezafungin, including comparisons with different dosing regimens and the use of additional fungal strains, for the treatment of aspergillosis caused by azole-resistant *Aspergillus*.
